# The role of FDG-PET imaging as a prognostic marker of outcome in primary mediastinal B-cell lymphoma

**DOI:** 10.1002/cam4.322

**Published:** 2014-09-10

**Authors:** Sarah J Nagle, Elise A Chong, Seble Chekol, Nirav N Shah, Sunita D Nasta, Eli Glatstein, John P Plastaras, Drew A Torigian, Stephen J Schuster, Jakub Svoboda

**Affiliations:** 1 Lymphoma Program, Abramson Cancer Center, University of PennsylvaniaPhiladelphia, Pennsylvania; 2 Department of Pathology and Laboratory Medicine, University of PennsylvaniaPhiladelphia, Pennsylvania; 3 Department of Radiation Oncology, University of PennsylvaniaPhiladelphia, Pennsylvania; 4 Department of Radiology, University of PennsylvaniaPhiladelphia, Pennsylvania

**Keywords:** Non-Hodgkin lymphoma, positron emission tomography, prognosis, R-CHOP protocol, treatment outcome

## Abstract

Primary mediastinal B-cell lymphoma (PMBL) is a subtype of diffuse large B-cell lymphoma (DLBCL) that arises in the mediastinum from B-cells of thymic origin. Optimal management of patients with PMBL remains controversial. The present study evaluates outcomes of 27 PMBL patients treated with R-CHOP with or without radiation therapy (RT). It investigates the role of both interim and posttreatment fluorodeoxyglucose-positron emission tomography (FDG-PET) as prognostic markers of outcome. Additionally, it assesses postprogression therapies in the six patients who had progressive disease. At a median follow-up of 41.5 months (range: 6.1–147.2 months), OS was 95.5% (95% CI = 71.9–99.4) and progression-free survival (PFS) was 70.4% (95% CI = 49.4–83.9) for the entire cohort. The negative predictive values of interim and posttreatment FDG-PET scans were both 100%. Patients who failed initial therapy and were treated with salvage regimens and autologous stem cell transplantation (ASCT) all achieved and maintained CR. PMBL patients can achieve excellent outcomes with minimal toxicities when treated with R-CHOP with or without RT. Negative interim and negative posttreatment FDG-PET results identified PMBL patients who achieve long-term remission. However, the significance of both positive interim and positive posttreatment FDG-PET results needs to be better defined. Those who failed initial therapy were successfully treated with salvage regimens and ASCT.

## Introduction

Primary mediastinal B-cell lymphoma (PMBL) is a distinct clinicopathologic subtype of diffuse large B-cell lymphoma (DLBCL) that arises from B cells of thymic origin. It represents less than 3% of all non-Hodgkin lymphomas (NHLs). It typically presents in young women in their 20s–30s with a rapidly expanding anterior mediastinal mass, ultimately resulting in local compressive effects 1. The optimal first-line treatment for PMBL remains controversial.

Historically, the standard treatment for DLBCL and its many subtypes was cyclophosphamide, doxorubicin, vincristine, and prednisone (CHOP) with the variable addition of radiation therapy (RT). Outcomes in patients with PMBL treated with this regimen were poor with event-free survival (EFS) and overall survival (OS) of only 34% and 51%, respectively 2. However, more recent data indicate that the addition of rituximab to the CHOP regimen (R-CHOP) significantly improves outcomes in PMBL patients, with one study finding a 5-year EFS and OS of 80% and 89%, respectively 3.

Efforts to further improve outcomes led to the use of aggressive chemotherapy regimens such as dose-adjusted rituximab, etoposide, prednisone, vincristine, cyclophosphamide, and doxorubicin (DA-EPOCH-R) in this setting 4. A recently published NCI phase 2 trial showed impressive results with EFS of 93% in a group of 51 PMBL patients. However, these results have not been confirmed in a larger cooperative study and there are no randomized trials comparing this regimen to R-CHOP in PMBL. Some concerns such as long-term toxicity, need for inpatient administration, and fertility issues with DA-EPOCH-R have been raised as well.

While R-CHOP with or without RT cures the majority of patients with PMBL, it is important to recognize early those patients who may be refractory to this regimen and may benefit from escalating to a more aggressive therapeutic approach. The International Prognostic Index (IPI), which is typically used as a predictor of outcome in DLBCL is of limited utility in PMBL due to the age distribution of this disease and its usual confinement to the mediastinum. As many patients with PMBL have low-IPI scores at presentation, this index may not be consistent with the patient's true prognosis 5. One possible tool to identify R-CHOP treatment failure early may be the tumor metabolic response based on [18F] fluorodeoxyglucose-positron emission tomography (FDG-PET) imaging.

FDG-PET has emerged as an important study in the diagnosis, staging, response assessment, and RT planning for aggressive NHL and Hodgkin lymphoma (HL). As compared with conventional computed tomography (CT), FDG-PET utilizes radiolabeled glucose to assess metabolic activity within tumors. It may distinguish between viable tumor and necrosis or fibrosis in a patient without other signs or symptoms of active disease 6. Early identification of refractory disease may provide patients with a basis for alternative treatment strategies. In the past decade, FDG-PET scanners are being combined with low intensity, noncontrast CT scanners and referred to as FDG-PET/CT. The FDG-PET images are acquired immediately after the CT is obtained. The fused images then allow for better anatomic localization of the lesions.

Interim restaging FDG-PET scans are highly predictive of outcome in patients with aggressive NHL and HL. This remains an area of active investigation with several ongoing clinical trials in HL utilizing response-adapted treatment algorithms 7–11. Additionally, multiple studies have demonstrated the utility of a posttreatment FDG-PET for response assessment in HL and aggressive NHL 6,12–16. However, the role of interim and posttreatment FDG-PET has not been well described in PMBL.

The purpose of the current study is to evaluate outcomes of PMBL patients treated with R-CHOP with or without RT and to investigate the role of both interim and posttreatment FDG-PET as prognostic markers.

## Methods

### Study design

We conducted a retrospective study using our institutional database of PMBL patients treated with first-line R-CHOP with or without RT. The study was approved by the institutional review board of the University of Pennsylvania.

### Patients

The patient population consisted of adult patients who were treated with R-CHOP with or without RT at the Hospital of the University of Pennsylvania between 1 January 2001 and 31 December 2013. In order to be included in the analysis, patients must have received an interim and/or posttreatment FDG-PET. Patients were excluded if they received prior therapy for PMBL, if they were less than 18 years of age at the time of diagnosis, or if they were pregnant at the time of diagnosis.

We identified 37 previously untreated patients with PMBL who underwent first-line therapy at our institution between 1 January 2001 and 31 December 2013. Seven patients were excluded from the analysis because they were treated with CHOP only, DA-EPOCH-R, or a nonanthracycline containing regimen. Thirty patients were treated with R-CHOP21 with or without RT. Three patients were excluded because they had not yet completed their treatment course by 31 December 2013 (*n* = 2) or because of pregnancy at the time of diagnosis (*n* = 1). The remaining 27 patients were further analyzed.

### FDG-PET examination

Patients were evaluated with an interim and/or a posttreatment FDG-PET. The interim FDG-PET occurred following the second and/or fourth cycle of R-CHOP and always occurred immediately before the subsequent treatment cycle to minimize false-positive results. The posttreatment FDG-PET was obtained at least 4 weeks following chemotherapy and prior to consolidative RT in patients who received RT. FDG-PET was graded as positive or negative based on the consensus criteria from the International Harmonization Project 17.

### Assessment of the entire cohort

The entire patient cohort was evaluated to assess response to therapy with R-CHOP with or without RT 18. OS was defined as the time from the date of initial treatment to the date of last follow-up or death from any cause. Progression-free survival (PFS) was calculated from the date of initial treatment until the date of disease progression or the date of last follow-up. Patients who had progressive disease (PD) were further evaluated for postprogression therapies and response to those therapies. Toxicities to R-CHOP with or without RT, based on the National Cancer Institute Common Terminology Criteria for Adverse Events (NCI CTCAE) Version 4, were also assessed and recorded 19.

### Assessment based on FDG-PET results

For the analysis examining the role of interim FDG-PET, the patients were labeled at “negative” if they achieved a metabolic complete response (CR) on any interim FDG-PET. Those who underwent FDG-PET but never achieved any negative interim scan were labeled as “positive.” OS and PFS were calculated for each of the following subgroups: positive versus negative interim FDG-PET and positive versus negative posttreatment FDG-PET. Patients with a positive posttreatment FDG-PET were identified and further assessed as to additional investigations and therapies. We also determined positive predictive value (PPV) and negative predictive value (NPV) for interim and posttreatment FDG-PET.

### Statistical analysis

PFS and OS were estimated using the Kaplan–Meier method. *P* ≤ 0.05 were considered statistically significant. Statistical analyses were performed using STATA version 12.1 (StataCorp, College Station, TX).

## Results

### Entire cohort

We identified 27 previously untreated patients with PMBL who were treated with R-CHOP with or without RT at our institution between 1 January 2001 and 31 December 2013. Nineteen of the 27 patients underwent consolidative RT following treatment with R-CHOP. The baseline characteristics at diagnosis are presented in Table1.

**Table 1:** Patient characteristics.

Age	
Median	36
Range	18–57
Sex	
Male	12
Female	15
Tumor bulk	
>10 cm	14
≤10 cm	13
Radiation therapy	
Yes	19
No	8
IPI at diagnosis	
0–1	23
2	2
≥3	2
Disease stage	
I	8
II	16
III	0
IV	3
Interim FDG-PET	
After two cycles	13
After four cycles	21
After both two and four cycles	11
Posttreatment FDG-PET	19

IPI, International Prognostic Index; FDG-PET, fluorodeoxyglucose-positron emission tomography.

**Figure 1:**
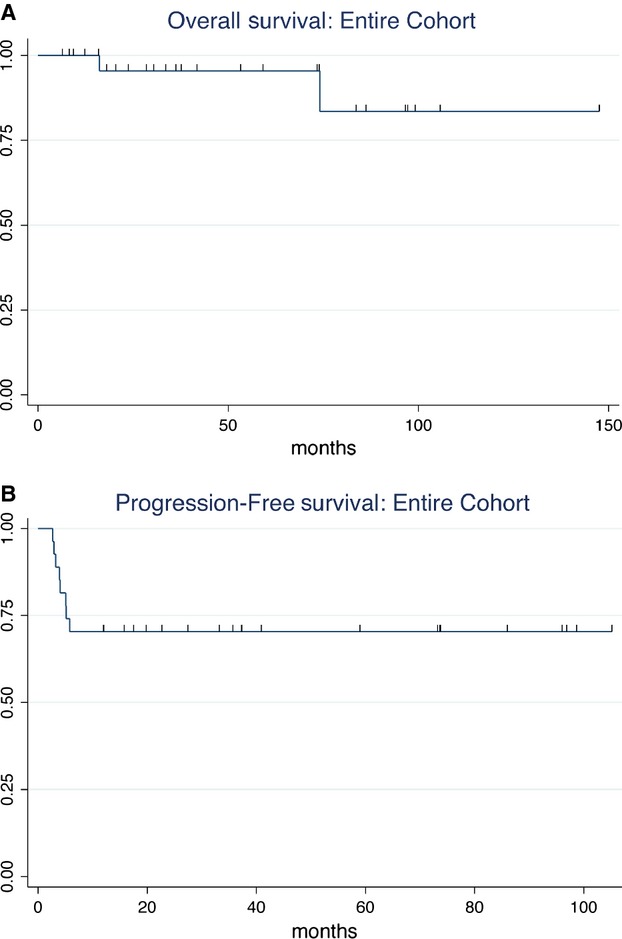
The Kaplan–Meier curves represent the (A) overall survival and (B) progression-free survival for the entire cohort.

At a median follow-up of 41.5 months (range = 6.1–147.2 months), OS was 95.5% (95% CI = 71.9–99.4) and PFS was 70.4% (95% CI = 49.4–83.9) for the entire cohort (Fig.1). The OS at 1 year was 100%.

Nineteen of the 27 patients received consolidative RT. Of these patients, eight had a positive interim FDG-PET, seven had a negative interim FDG-PET, and four did not undergo an interim FDG-PET scan. A total of three patients had a positive posttreatment FDG-PET, eight had a negative FDG-PET, and eight did not undergo a posttreatment FDG-PET scan. All eight of the patients who did not receive consolidative RT had a positive interim FDG-PET. The results of post-treatment FDG-PET in this group were as follows: three patients with a positive FDG-PET, one patient with a negative FDG-PET, and four patients who did not undergo a posttreatment FDG-PET scan. The decision to use consolidative RT was solely based on the preference of the treating physician and did not correlate with FDG-PET status at the interim or posttreatment scan. However, those patients (*N* = 5) who progressed on systemic chemotherapy did not receive RT.

Eight of the 27 patients had PD following treatment with R-CHOP with or without RT. Seven of the 8 patients progressed during or immediately following treatment with R-CHOP, prior to RT. The outcomes of the patients who had PD were as follows: one patient was treated with methotrexate, rituximab, and temozolomide for a central nervous system relapse and achieved CR; one patient was treated with rituximab, ifosfamide, carboplatin, and etoposide (R-ICE) followed by rituximab and bendamustine, but had disease progression and died; five patients underwent salvage chemotherapy with R-ICE followed by high-dose chemotherapy (HDT) and autologous stem cell transplantation (ASCT) and all achieved CR. At the time of analysis, all five patients who underwent ASCT are alive without active disease. At the time of analysis, one patient was undergoing HDT in preparation for ASCT. Only 1 of 27 patients died of progressive PMBL. One patient died of accidental causes unrelated to PMBL and without evidence of active disease.

Notable toxicities of the entire cohort of 27 patients were as follows: 2 (7%) patients developed grade 2 radiation pneumonitis, 3 (11%) patients were treated for grade 3 neutropenic fever, 4 (15%) patients developed grade 2 hypothyroidism, and 3 (11%) patients had peripheral sensory neuropathy (grade 1, *n* = 2; grade 2, *n* = 1).

### Results based on interim FDG-PET

Twenty-three (85%) patients underwent an interim FDG-PET. Thirteen patients had imaging after two cycles of R-CHOP, 21 patients had imaging after four cycles of R-CHOP, and 11 patients had an FDG-PET at both time points. Twenty-two patients underwent FDG-PET/CT and one patient underwent FDG-PET without CT. Four (15%) patients did not undergo an interim FDG-PET scan due to physician preference.

Following two cycles of R-CHOP, 11 of the 13 patients (85%) who underwent FDG-PET had a positive scan and 2 of the 13 patients (15%) had a negative scan. Of the two patients who had a negative scan, one was repeated after the fourth cycle of R-CHOP and was again negative; the FDG-PET was not repeated in the other patient. Neither patient who had a negative FDG-PET after the second cycle of R-CHOP relapsed or had PD. The 11 patients who had a positive scan following the second cycle of R-CHOP had heterogeneous outcomes. Ten patients underwent a repeat FDG-PET following the fourth cycle of R-CHOP. Two of the 10 scans (20%) were negative and the patients have not relapsed. Eight of the 10 scans (80%) were positive. Five of the eight patients with a positive scan (63%) had PD and required further therapy, whereas three of the eight patients with a positive scan (38%) did not relapse or have PD. One patient with a positive FDG-PET following the second cycle of R-CHOP did not have a repeat FDG-PET following the fourth cycle, but did have evidence of PD on his posttreatment FDG-PET and required further therapy.

**Figure 2:**
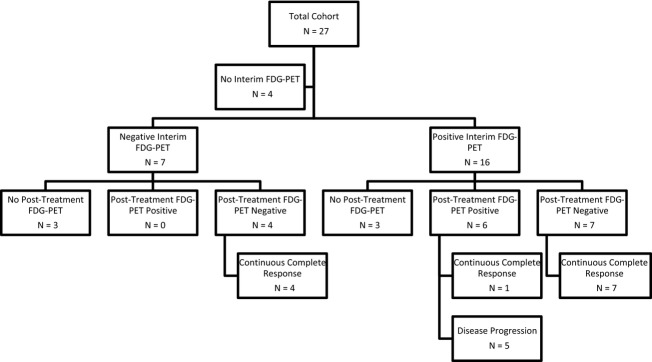
Summary of clinical outcome according to interim FDG-PET results. FDG-PET, fluorodeoxyglucose-positron emission tomography.

Following four cycles of R-CHOP, 21 patients underwent FDG-PET. Fifteen of the 21 patients (71%) had a positive FDG-PET at this time point and six of the 21 patients (29%) had a negative FDG-PET. Of the six patients with a negative FDG-PET following the fourth cycle of R-CHOP, none relapsed or had PD. Of the 15 patients with a positive FDG-PET following the fourth cycle of R-CHOP, eight patients (53%) did not relapse or have PD and seven of the 15 patients (47%) did have PD requiring further therapy (Fig.2).

Seven of the 23 patients (30%) who underwent FDG-PET after the second or fourth cycle of R-CHOP had a negative scan at one or both time points. None of the patients who had a negative FDG-PET following either or both cycles of R-CHOP relapsed or had PD.

**Figure 3:**
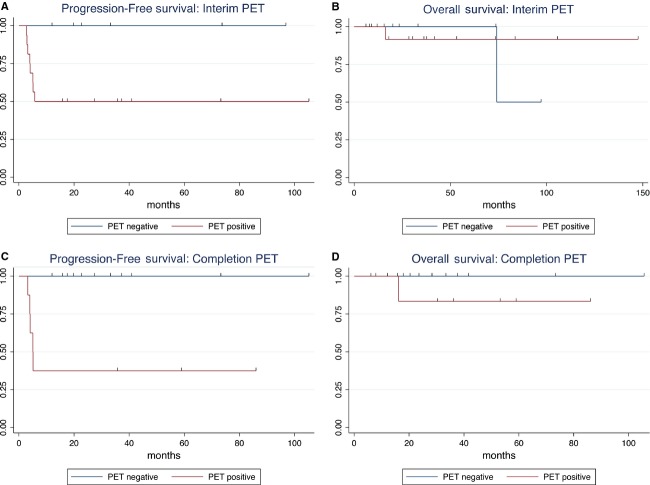
Survival analysis according to interim and posttreatment FDG-PET results. The blue line represents a negative FDG-PET and the red line represents a positive FDG-PET. The results shown are for (A) progression-free survival based on interim FDG-PET, (B) overall survival based on interim FDG-PET, (C) progression-free survival based on posttreatment FDG-PET, and (D) overall survival based on FDG-PET. FDG-PET, fluorodeoxyglucose-positron emission tomography.

The median PFS for patients with a positive interim FDG-PET was significantly less than patients with a negative FDG-PET (5.77 months vs. not yet reached at a median follow-up of 33.2 months; *P* = 0.032) (Fig.3A). There was no difference in OS between patients who had a positive interim FDG-PET and those who had a negative interim FDG-PET (*P* = 0.69) (Fig.3B). One patient with a negative interim FDG-PET suffered an accidental death unrelated to PMBL and one patient with a positive interim FDG-PET died of progressive PMBL. The NPV of an interim FDG-PET was 100% and the PPV was 50%.

### Results based on posttreatment FDG-PET

Nineteen patients (70%) underwent a posttreatment FDG-PET, all with CT. Eight patients (30%) did not undergo a posttreatment FDG-PET for heterogeneous reasons. Three of the eight patients had a negative interim FDG-PET and were subsequently followed by CT scans. Two patients who did not have a negative interim FDG-PET were also followed by CT scans and achieved a complete anatomic response; therefore FDG-PET was not felt to be necessary by the treating physician. The remaining three patients had PD that was identified clinically prior to evaluation by a completion FDG-PET scan.

Eleven of the 19 patients (58%) had a negative posttreatment FDG-PET and did not relapse. Eight of the 19 patients (42%) had a positive posttreatment FDG-PET. The patients with a positive posttreatment FDG-PET had the following outcomes: one patient underwent a biopsy that was negative and has not relapsed; two patients had sequential FDG-PET scans until resolution of their abnormality and have not relapsed; five patients were treated with salvage therapy.

All five patients who had a positive FDG-PET and underwent salvage therapy did not have PD that was suspected clinically. PD in each patient was found on posttreatment imaging and salvage treatment was initiated at that time. Of the five patients who were treated with salvage therapy, three patients had a sustained CR, one patient died of PD, and one patient was in preparation to undergo ASCT at the time of analysis.

There was a significant difference in PFS between patients who had a negative posttreatment FDG-PET and those who had a positive scan at this time point (5.0 months vs. not yet reached at a median follow-up of 33.2 months; *P* = 0.0021) (Fig.3C). The OS was not significantly different for patients with a positive posttreatment FDG-PET versus those with a negative posttreatment FDG-PET (*P* = 0.22) (Fig.3D). Only one patient with a positive posttreatment FDG-PET died of progressive PMBL, and there were no deaths in patients with a negative posttreatment FDG-PET. The NPV for a posttreatment FDG-PET was 100% and the PPV was 63%.

## Discussion

The present study shows excellent outcomes in patients with PMBL treated with R-CHOP with or without RT with minimal toxicities. At a median follow-up of 41.5 months, OS was 95.5% and PFS was 70.4% for the entire cohort. This is consistent with other studies, suggesting that the vast majority of patients with PMBL can be successfully treated with R-CHOP with or without RT 3,20–22. However, there is a subgroup of patients who are refractory to this therapeutic approach. This has led to trials of more aggressive chemotherapy regimens such as DA-EPOCH-R in PMBL patients 4. It is unclear, however, which patients benefit from DA-EPOCH-R and who will be successfully treated with R-CHOP with or without RT. One potential early prognostic marker that this study investigated is metabolic response based on interim and posttreatment FDG-PET.

There is emerging data regarding the role of both interim and posttreatment FDG-PET for predicting outcomes in patients various lymphoma subtypes. Studies have shown correlation between an early decline in tumor FDG uptake and outcome in patients with aggressive NHL and HL, suggesting that interim FDG-PET offers the opportunity to predict outcomes and alter management. Kostakoglu et al. 23 performed FDG-PET after one cycle of chemotherapy in 47 patients with DLBCL or HL. All of the patients who had a negative FDG-PET scan after one cycle of chemotherapy had a sustained CR with a median follow-up of 28 months. Conversely, 14 of the 16 patients with a positive FDG-PET at this time point relapsed or had PD. Other studies in patients with both aggressive NHL and HL have supported these findings 7,23–25. Several recent clinical trials have been designed to use the results of interim FDG-PET as response-adapted strategy and to investigate if escalating or de-escalating therapy may improve outcomes.

Posttreatment FDG-PET is an important prognostic marker for patients with HL and aggressive NHL 13,15,26. The sensitivity and specificity of FDG-PET for the detection of residual disease after completion of first-line therapy are 84% and 90%, respectively, for HL and 72% and 100%, respectively, for aggressive NHL 12. Spaepen et al. 13 evaluated 93 patients with NHL with FDG-PET after the completion of first-line therapy. Fifty-six of 67 patients with a negative FDG-PET remained in CR and only 11 of these patients relapsed or had refractory disease. Conversely 26 patients had a positive FDG-PET at the completion of therapy and all relapsed or had PD.

Despite the utility of interim and posttreatment FDG-PET in HL and aggressive NHL, there is a paucity of data regarding the role of FDG-PET specifically in PMBL. Ceriani et al. 27 prospectively evaluated the role of PET/CT after rituximab and anthrocycline-containing regimens in patients with PMBL and found that a negative PET/CT at completion of treatment was associated with a longer PFS. Our data suggests that interim and posttreatment FDG-PET in patients with PMBL can help predict outcome. Patients who had a negative scan after two or four cycles of R-CHOP, achieved a CR and did not relapse. However, the outcome of patients who had a positive interim scan was variable. Although 50% of the patients who had a positive interim FDG-PET relapsed or had PD, 50% of patients who had a positive interim FDG-PET achieved and maintained CR at 1 year after treatment. Therefore, while a negative FDG-PET after two or four cycles of chemotherapy may predict excellent outcome in PMBL patients, the clinical significance of a positive interim FDG-PET and the possible advantage of changing therapy in this patient group remains unclear and will need to be studied before routinely implemented in clinical practice.

In addition, our data suggest that a negative posttreatment FDG-PET is a good predictor of outcome. In the present study, a negative posttreatment FDG-PET had a predictive value of 100% in PMBL, consistent with other recent studies that evaluated the use of posttreatment FDG-PET in patients with PMBL 4. With a median follow-up of 41.5 months, there were no recorded relapses in patients who had a negative posttreatment FDG-PET. The utility of a positive FDG-PET remains uncertain in this setting. Five (63%) of the eight patients who had a positive posttreatment FDG-PET had PD. However, three of the eight patients (38%) with a positive posttreatment FDG-PET achieved and maintained CR. Two of these three patients were followed with sequential FDG-PET until resolution of FDG avidity; the third underwent a biopsy, which was negative. Therefore, a positive FDG-PET following completion of therapy requires further evaluation, tissue biopsy to confirm the diagnosis, or very close follow-up in order to and develop an optimal treatment plan.

There are multiple potential challenges to FDG-PET interpretation, particularly in PMBL patients. False-positive results can occur secondary to physiologic uptake by normal tissues, inflammatory processes and thymic rebound hyperplasia following chemoimmunotherapy. FDG-PET should be delayed for at least 2 weeks after administration of chemotherapy and 2 months after RT. This was accomplished in our patient population, decreasing the potential for false-positive results. Despite this, false-positive results still were observed. This explains our finding that despite the excellent NPV of FDG-PET for residual disease in PMBL, the PPV is poor and a positive FDG-PET may or may not represent residual disease 28. One additional limitation of the present study is the use of the traditional IHP criteria for both interim and posttreatment scans rather than the more recently applied Deauville criteria 29.

In our study, patients who progressed received salvage chemotherapy followed by HDT and ASCT, and all achieved and maintained CR. Excellent results of salvage chemotherapy and ASCT in our PMBL cohort could be explained by the ability of FDG-PET to identify patients with refractory or relapsed disease early. We speculate that this may lead to shortening of the time lapsed between the first-line therapy and salvage which may have resulted in better outcomes than previously reported.

## Conclusions

The present study reports outcomes of PMBL patients treated with R-CHOP with or without RT and investigates the role of both interim and posttreatment FDG-PET to provide prognostic markers of outcome. We found that PMBL patients can achieve excellent outcomes with minimal toxicities when using this treatment strategy. Those who relapsed were successfully salvaged by ASCT. Negative interim and negative posttreatment FDG-PET results identify PMBL patients who achieve long-term remission. However, the significance of positive interim and of positive posttreatment FDG-PET in this setting needs to be better defined.

## References

[b1] Johnson PW, Davies AJ (2008). Primary mediastinal B-cell lymphoma. Hematology Am. Soc. Hematol. Educ. Program.

[b2] Hamlin PA, Portlock CS, Straus DJ, Noy A, Singer A, Horwitz SM (2005). Primary mediastinal large B-cell lymphoma: optimal therapy and prognostic factor analysis in 141 consecutive patients treated at Memorial Sloan Kettering from 1980 to 1999. Br. J. Haematol.

[b3] Vassilakopoulos TP, Pangalis GA, Katsigiannis A, Papageorgiou SG, Constantinou N, Terpos E (2012). Rituximab, cyclophosphamide, doxorubicin, vincristine, and prednisone with or without radiotherapy in primary mediastinal large B-cell lymphoma: the emerging standard of care. Oncologist.

[b4] Dunleavy K, Pittaluga S, Maeda LS, Advani R, Chen CC, Hessler J (2013). Dose-adjusted EPOCH-rituximab therapy in primary mediastinal B-cell lymphoma. N. Engl. J. Med.

[b5] Abou-Elella AA, Weisenburger DD, Vose JM, Kollath JP, Bast MA, Bierman PJ (1999). Primary mediastinal large B-cell lymphoma: a clinicopathologic study of 43 patients from the Nebraska Lymphoma Study Group. J. Clin. Oncol.

[b6] Juweid ME, Cheson BD (2005). Role of positron emission tomography in lymphoma. J. Clin. Oncol.

[b7] Spaepen K, Stroobants S, Dupont P, Vandenberghe P, Thomas J, de Groot T (2002). Early restaging positron emission tomography with (18)F-fluorodeoxyglucose predicts outcome in patients with aggressive non-Hodgkin's lymphoma. Ann. Oncol.

[b8] Romer W, Hanauske AR, Ziegler S, Thodtmann R, Weber W, Fuchs C (1998). Positron emission tomography in non-Hodgkin's lymphoma: assessment of chemotherapy with fluorodeoxyglucose. Blood.

[b9] Raemaekers JM, Andre MP, Federico M, Girinsky T, Oumedaly R, Brusamolino E (2014). Omitting radiotherapy in early positron emission tomography-negative stage I/II Hodgkin lymphoma is associated with an increased risk of early relapse: clinical results of the preplanned interim analysis of the randomized EORTC/LYSA/FIL H10 trial. J. Clin. Oncol.

[b10] Kostakoglu L, Coleman M, Leonard JP, Kuji I, Zoe H, Goldsmith SJ (2002). PET predicts prognosis after 1 cycle of chemotherapy in aggressive lymphoma and Hodgkin's disease. J. Nucl. Med.

[b11] Zinzani PL, Gandolfi L, Broccoli A, Argnani L, Fanti S, Pellegrini C (2011). Midtreatment 18F-fluorodeoxyglucose positron-emission tomography in aggressive non-Hodgkin lymphoma. Cancer.

[b12] Zijlstra JM, Lindauer-van der Werf G, Hoekstra OS, Hooft L, Riphagen II, Huijgens PC (2006). 18F-fluoro-deoxyglucose positron emission tomography for post-treatment evaluation of malignant lymphoma: a systematic review. Haematologica.

[b13] Spaepen K, Stroobants S, Dupont P, Van Steenweghen S, Thomas J, Vandenberghe P (2001). Prognostic value of positron emission tomography (PET) with fluorine-18 fluorodeoxyglucose ([18F]FDG) after first-line chemotherapy in non-Hodgkin's lymphoma: Is [18F]FDG-PET a valid alternative to conventional diagnostic methods?. J. Clin. Oncol.

[b14] Weihrauch MR, Re D, Scheidhauer K, Ansen S, Dietlein M, Bischoff S (2001). Thoracic positron emission tomography using 18F-fluorodeoxyglucose for the evaluation of residual mediastinal Hodgkin disease. Blood.

[b15] Naumann R, Vaic A, Beuthien-Baumann B, Bredow J, Kropp J, Kittner T (2001). Prognostic value of positron emission tomography in the evaluation of post-treatment residual mass in patients with Hodgkin's disease and non-Hodgkin's lymphoma. Br. J. Haematol.

[b16] Jerusalem G, Beguin Y, Fassotte MF, Najjar F, Paulus P, Rigo P (1999). Whole-body positron emission tomography using 18F-fluorodeoxyglucose for posttreatment evaluation in Hodgkin's disease and non-Hodgkin's lymphoma has higher diagnostic and prognostic value than classical computed tomography scan imaging. Blood.

[b17] Juweid ME, Stroobants S, Hoekstra OS, Mottaghy FM, Dietlein M, Guermazi A (2007). Use of positron emission tomography for response assessment of lymphoma: consensus of the imaging subcommittee of International Harmonization Project in Lymphoma. J. Clin. Oncol.

[b18] Cheson BD, Pfistner B, Juweid ME, Gascoyne RD, Specht L, Horning SJ (2007). Revised response criteria for malignant lymphoma. J. Clin. Oncol.

[b19] National Cancer Institute (2009).

[b20] Savage KJ, Al-Rajhi N, Voss N, Paltiel C, Klasa R, Gascoyne RD (2006). Favorable outcome of primary mediastinal large B-cell lymphoma in a single institution: the British Columbia experience. Ann. Oncol.

[b21] Ahn HK, Kim SJ, Yun J, Yi JH, Kim JH, Won YW (2010). Improved treatment outcome of primary mediastinal large B-cell lymphoma after introduction of rituximab in Korean patients. Int. J. Hematol.

[b22] Pfreundschuh M, Trumper L, Osterborg A, Pettengell R, Trneny M, Imrie K (2006). CHOP-like chemotherapy plus rituximab versus CHOP-like chemotherapy alone in young patients with good-prognosis diffuse large-B-cell lymphoma: a randomised controlled trial by the MabThera international trial (MInT) group. Lancet Oncol.

[b23] Kostakoglu L, Goldsmith SJ, Leonard JP, Christos P, Furman RR, Atasever T (2006). FDG-PET after 1 cycle of therapy predicts outcome in diffuse large cell lymphoma and classic Hodgkin disease. Cancer.

[b24] Safar V, Dupuis J, Itti E, Jardin F, Fruchart C, Bardet S (2012). Interim [18F]fluorodeoxyglucose positron emission tomography scan in diffuse large B-cell lymphoma treated with anthracycline-based chemotherapy plus rituximab. J. Clin. Oncol.

[b25] Hutchings M, Loft A, Hansen M, Pedersen LM, Berthelsen AK, Keiding S (2006). Position emission tomography with or without computed tomography in the primary staging of Hodgkin's lymphoma. Haematologica.

[b26] Zinzani PL, Fanti S, Battista G, Tani M, Castellucci P, Stefoni V (2004). Predictive role of positron emission tomography (PET) in the outcome of lymphoma patients. Br. J. Cancer.

[b27] Ceriani L, Zucca E, Zinzani PL, Ferreri AJ, Vitolo U, Stelitano C (2012). Role of positron emission tomography (PET/CT) in primary mediastinal large B cell lymphoma (PMLBCL): preliminary results of an international phase II trial (IELSG-26 study) conducted on behalf of the International Extranodal Lymphoma Study Group (IELSG), the fondazione italiana linfomi (FIL) and the UK NCRI lymphoma group. Blood.

[b28] Moskowitz CH, Schoder H, Teruya-Feldstein J, Sima C, Iasonos A, Portlock CS (2010). Risk-adapted dose-dense immunochemotherapy determined by interim FDG-PET in advanced-stage diffuse large B-cell lymphoma. J. Clin. Oncol.

[b29] Moskowitz CH (2012). Interim PET-CT in the management of diffuse large B-cell lymphoma. Hematology Am. Soc. Hematol. Educ. Program.

